# A Megabase-Scale Deletion is Associated with Phenotypic Variation of Multiple Traits in Maize

**DOI:** 10.1534/genetics.118.301567

**Published:** 2018-11-01

**Authors:** Xuesong Han, Yao Qin, Feng Yu, Xuemei Ren, Zuxin Zhang, Fazhan Qiu

**Affiliations:** National Key Laboratory of Crop Genetic Improvement, Huazhong Agricultural University, Wuhan 430070, P.R. China

**Keywords:** *Zea mays* L., megabase-scale deletion, pleiotropy, functional redundancy

## Abstract

Genomic deletions are pervasive in the maize (*Zea mays* L.) genome, and play important roles in phenotypic variation and adaptive evolution. However, little is known about the biological functions of these genomic deletions. Here, we report the biological function of a megabase-scale deletion, which we identified by position-based cloning of the *multi-trait weakened* (*muw*) mutant, which is inherited as a single recessive locus. *MUW* was mapped to a 5.16-Mb region on chromosome 2. The 5.16-Mb deletion in the *muw* mutant led to the loss of 48 genes and was responsible for a set of phenotypic abnormities, including wilting leaves, poor yield performance, reduced plant height, increased stomatal density, and rapid water loss. While *muw* appears to have resulted from double-stranded break repair that was not dependent on intragenomic DNA homology, extensive duplication of maize genes may have mitigated its effects and facilitated its survival.

GENOMIC structural variations (SVs), including kilobase- to megabase-sized deletions, insertions, inversions, and translocations (Feuk *et al.* 2006), are brought about fundamentally by chromosomal events, such as transposition of transposable elements (TEs), DNA recombination, replication, and repair-associated processes (Morgante *et al.* 2005; Hastings *et al.* 2009), which lead to genomic rearrangement, expansion, and reduction. These large-scale genomic variations are also thought to make important contributions to genetic diversity and phenotypic variation in living organisms (Sebat 2007; Hurles *et al.* 2008; Springer *et al.* 2009). Large-scale SVs, including genomic deletions, were first identified through cytogenetic studies in maize, which affect intraspecific collinearity (Fu and Dooner 2002; Brunner *et al.* 2005). Over the past 10 years, sequence-based methodologies have identified numerous SVs in the maize genome (Saxena *et al.* 2014; Sun *et al.* 2018; Zhang *et al.* 2018). Among them, genomic deletion is one of the most abundant SV types and affects a significant portion of the maize genome (Springer *et al.* 2009; Lai *et al.* 2010; Chia *et al.* 2012; Lu *et al.* 2015; Jiao *et al.* 2017; Sun *et al.* 2018).

Increasing sequencing data have demonstrated that genomic deletions that contain genes and regulatory regions usually have a significant effect on fitness and domestication. During maize domestication, some teosinte genome regions have been selectively lost in response to habitat-specific selective pressures and human needs. For example, the genome of landrace *Palomero Toluqueño* is ∼22% smaller than that of B73, especially due to a lesser amount of DNA repeats (Vielle-Calzada *et al.* 2009). In other words, the expansion of the B73 genome is primarily caused by those increased DNA repeats. Among modern cultivated varieties, a large number of genomic deletions are also found. For example, Fu and Dooner (2002) have sequenced the Bacterial Artificial Chromosome (BAC) harboring *bronze* (*bz*) of B73 and McC inbred lines, and found obvious differences between both inbred lines in the number and position of retrotransposons, with ∼40 kb of retrotransposon sequences absent in McC but present in B73. In particular, genes encoded in the 150-kb region are different in the two inbred lines. Among those genes, six genes are shared by B73 and McC, but four genes are lost in B73, showing loss of micro-collinearity at the DNA level (Fu and Dooner 2002). Genomic deletions also give rise to universal genetic effects. In rice (*Oryza sativa*), a 38.3-kb fragment harboring *Grain number*, *plant height and heading date7* (*Ghd7*), which has a large effect on heading date and yield-related traits, is completely deleted in Zhenshan 97 but is present in Minghui 63 (Xue *et al.* 2008). A 254-kb genomic deletion reduces palmitic acid level in soybean (*Glycine max* L.) seeds (Goettel *et al.* 2016). Moreover, a 147-kb deletion containing maize *wall-associated kinase* (*ZmWAK*) in the major *head smut quantitative resistance locus* (named as *qHSR1*) confers susceptibility to the fungal disease head smut (Zuo *et al.* 2015). Additionally, genome-wide association studies (GWAS) show that SVs, including genomic deletions, are enriched at those loci associated with important traits, such as disease resistance, flowering time, plant height, and leaf-related traits in maize (Chia *et al.* 2012; Lu *et al.* 2015). These investigations provide vital links between genomic regions and phenotypes, and are shaping our understanding on the impact of genomic deletions as a major cause of plant phenotypic variation. Although massive genomic deletions have been identified by whole-genome sequencing in maize, little is known about the biological functions of these large-scale deletions.

To infer the potential mechanism of origin of genomic deletion, breakpoint junction sequences of massive genomic deletions have been detected from high-throughput sequencing data and been analyzed (Kidd *et al.* 2010; Jennes *et al.* 2011; Parks *et al.* 2015), and these investigations result in three hypotheses: nonallelic homologous recombination (NAHR), nonhomologous end joining (NHEJ), and microhomology-mediated replication-dependent recombination (MMRDR) (Chen *et al.* 2010; Jennes *et al.* 2011; Ottaviani *et al.* 2014). However, the molecular mechanism on the production of genomic deletions is still poorly understood in maize, except for the *Ac*/*Ds* transposable element system (Zhang and Peterson 1999). Moreover, homologous pairing of a large intercalary deletion and a normal complete homolog in heterozygotes leads to the formation of a deletion loop (Moustakas *et al.* 2006), causing severe suppression of recombination. During the process of fine mapping of QTL for quantitative traits in plant species, dramatic recombination suppression caused by chromosomal deletion is observed in the target interval. The suppression of recombination hinders narrowing the target interval by position-based cloning strategy, for example, the *Ghd7* locus in rice (Xue *et al.* 2008) and *qHSR1* locus in maize (Zuo *et al.* 2015).

The aim of the present study was to identify the causal mutation for the *muw* mutant by position-based cloning. We demonstrate that the causal mutation is a 5.16-Mb genomic deletion on chromosome 2 that contains 48 genes. We also report the biological function of the 5.16-Mb in the *muw* mutant, showing that this deletion induces a set of phenotypic abnormities, including wilting, decreased yield, reduced plant height, increased stomatal density, and accelerated water loss.

## Materials and Methods

### Plant materials

The maize spontaneous mutant *muw* with abnormalities in multiple characteristics was isolated from the maize Lian87 inbred line and crossed to another wild-type inbred line, V54, to develop the mapping population. To evaluate the effect of the *muw* mutation in different genetic backgrounds, the *muw* mutant was crossed to six maize inbred lines: B73, Mo17, 18599, Huangzaosi (HZS), HLZY, and Zong3. Among them, B73, HLZY, and Lian 87 are three inbred lines in Stiff Stalk (SS), Mo17 belongs to Non-Stiff Stalk (NSS), HZS belongs to Tang Si Ping Tou (TSPT), and Zong3 and 18599 are two inbred lines from mixed origins, respectively. The progenies of the six cross combinations were selfed separately to produce F_2_ individuals. To calculate genetic distance and recombination rate in the mapping region, two F_2_ populations, comprising 960 and 960 F_2_-individuals, respectively, were derived from the V54 × *muw* mutant and V54 × Lian87.

### Water loss and chlorophyll efflux assay

To measure the rate of water loss, the second leaf from the top of the *muw* and wild-type plants (Lian87) at the seven-leaf seedling stage were collected and weighed, and then weighed once every 30 min for 6 hr at room temperature (26°). The water-loss rate of the leaf was defined as the weight of water lost from the leaf at a specific time divided by the fresh weight (FW) with three replicates. Twelve leaves together were used for each measurement in three replicates.

The chlorophyll efflux assay was performed by measuring the permeability of the cuticle according to the description of Lolle *et al.* (1997) with minor modifications. Briefly, the second leaf from the top of *muw* and wild-type plants at the five-leaf seedling stage was collected and immersed in 80% ethanol in 50-ml tubes. These tubes were agitated on a shaker at 100 rpm. The chlorophyll in the supernatant was quantified using a UV 1800 spectrophotometer (Shimadzu, Japan) at wavelengths of 664 and 647 nm at six time points (0.5, 2, 4, 7, 12, and 24 hr) after initial immersion with three biological replicates. The chlorophyll efflux was expressed as a percentage of the chlorophyll amount at different time points to the total chlorophyll amount extracted after 24 hr of immersion.

### Measurement of photosynthetic pigments

The measurement of photosynthetic pigments was performed according to the description of Arnon (1949) and Lichtenthaler (1987). Briefly, 200 mg FW of ear leaves at the filling stage were sliced and then collected into a 50-ml tube, followed by adding 25 ml of 95% ethanol, and the tubes were placed in the dark for 12 hr. The light absorption values of the extracts were measured at the wavelengths of 665, 649, and 470 nm by a UV 1800 spectrophotometer with three biological replicates, each replicate consisted of 12 plants from the *muw* mutant and wild type. Chlorophyll a (Chl.a, mg/g FW), chlorophyll b (Chl.b, mg/g FW), and carotenoid concentration (Car., mg/g FW) were then calculated by the equations published by Lichtenthaler and Wellburn (1983):

Chl.a(mg/gFW)=13.96×A665−6.88×A649

Chl.b(mg/gFW)=24.96×A649−7.32×A665

Car.(mg/gFW)=(1000×A470−2.05×Chl.a−114.8×Chl.b)/245

### Abscisic acid (ABA) measurement and ABA response

Leaves (0.5 g) of *muw* and wild-type seedlings were collected, frozen in liquid nitrogen, and ground into fine powders, which were then collected into a centrifuge tube containing 4.5 ml of sample extraction buffer (Sun *et al.* 2017). The samples were lightly shaken overnight at 4° and then centrifuged at 4° for 10 min at 10,000 rpm. The supernatant was transferred into a new tube for measuring the concentration of ABA with an ABA ELISA kit (CUSABIO, College Park, MD).

To characterize the response of the *muw* mutant to ABA, we performed a germination assay as previously described with some modifications (Brugière *et al.* 2017). For *muw* mutant or Lian87 wild type, 100 seeds were soaked for 24 hr in sterile water (control) and 200 μM ABA (treatment) (Sigma, Santa Clara, CA), respectively, and soaked seeds were then germinated on filter papers wetted with sterile water. Germinated seeds were scored after 2–5 days, and germination rates of seeds under the control and ABA treatment were counted with three replicates.

### Cytological observations

Ear-leaves were sampled from the *muw* and wild-type plants at the filling stage for measuring stomata number. The lower epidermis of the leaf was peeled off using tweezers and placed onto a microscope slide according to the description of Muir *et al.* (2014). Stomata were counted from five microscopic fields of the middle zone of the leaf. The stomata number per unit area of the *muw* and wild-type leaves was compared.

Stem internodes from the *muw* and wild-type plants were fixed in 4% paraformaldehyde (Sigma) overnight. The fixed tissue samples were dehydrated in a graded series of ethanol (30, 50, 70, 85, 95, and 100% ethanol), embedded in Paraplast Plus (Sigma), then sectioned into 8-µm slices using a Leica RM2265 microtome (Leica Microsystems, Wetzlar, Hesse-Darmstadt, Germany). The slices were stained using 0.5% toluidine blue and subsequently photographed using a Leica MZFLIII microscope (Leica Microsystems). The vascular bundles and vessels in the largest vascular bundle were counted from the photographed images.

### Phenotyping agronomic traits

Four kernel traits, including 100-kernel weight (HKW), 20-kernel length (KL), 20-kernel width (KW), and 20-kernel thickness (KT), were measured using 60 *muw* mutant plants and 60 Lian87 wild-type plants with three repeated measurements. Four ear traits, including ear diameter (ED), cob diameter (CD), ear length (EL), and kernel row number (KRN), as well as plant height (PH) and ear height (EH) were also phenotyped in 60 *muw* mutant plants and 60 Lian87 wild-type plants.

### Map-based cloning of the *muw* mutation and molecular marker development

To clone the *muw* mutation, we crossed the *muw* mutant to the V54 inbred line to create the mapping population. Primary mapping of the *muw* mutation was performed using bulk-segregant analysis (BSA). Thirty *muw* and 30 wild-type F_2_ individuals were separately pooled and then screened using ∼1000 pairs of SSR markers that are chosen from the maize genome database (http://www.maizegdb.org/) and are distributed uniformly throughout the maize genome. For fine mapping, 14,496 F_2_ individuals were genotyped using markers flanking and locating in the mapping interval. Additionally, a total of 960 F_2_ individuals from each of two F_2_ populations (V54 × the *muw* mutant and V54 × Lian87) were genotyped with markers flanking the *muw* mutation to evaluate the recombination rate.

According to the BSA result, additional molecular markers (Supplemental Material, Table S1) were designed to narrow down the mapping region. The DNA sequences within the *umc1485*–*umc1635* interval on chromosome 2 were retrieved from the B73 genome sequence (RefGen_V4) (www.maizegdb.org) and were searched for simple sequence repeats (SSRs) using the SSRHunter software (Li and Wan 2005). All sequences covering SSRs were then used for searching against the B73 reference genome; only those unique sequences were selected for future marker design. Meanwhile, flanking sequences of small insertion/deletion (InDel) mutations (3–7 bp) in the *umc1485*–*umc1635* interval were downloaded from the Panzea website (www.panzea.org). Primer pairs were designed to generate PCR products of 100–200 bp using PrimerPlus version 4.0.0 (www.primer3plus.com) with the default settings. Mapmarker/exp3.0 software was used to reconstruct a linkage map (Lander *et al.* 1987).

### Detection of deletion breakpoints and PCR product sequencing

Position of deletion breakpoints was estimated roughly using PCR amplification. If one primer pair can amplify a PCR product that we expected in wild type but cannot in the *muw*, we suggest that the DNA segment is deleted in the *muw* genome. Therefore, we can estimate roughly the deletion interval, and those markers mapped in the deletion interval, in this way. The left breakpoint was located in L6 and L7, and the right breakpoint was located in R11 and R12. Primers that flanked the detected deletion region, L6-F and R12-R, were used to conduct PCR on *MUW* and *muw*. A specific PCR product of ∼12-kb that spanned the deletion suture point was amplified successfully in the *muw* mutant. The PCR product was then gel extracted and sequenced. Alignment of the PCR product sequence to the B73 V4 reference genome was performed to determine the exact breakpoint borders. The left and right breakpoints in the *MUW* were called at the edges of matching sequence. The deletion suture point in the *muw* was called where the broken ends are directly ligated.

The 12-kb amplicon was amplified by PCR from the genomic DNA of *muw* and sequenced on both strands with an ABI 3730 DNA analyzer (Tian Yi Hui Yuan Biotechnologies Co., Wuhan, China). For pairwise alignments and adenine and thymine contents analysis, two 2-kb segments near the left and right breakpoint were amplified by PCR from the genomic DNA of *MUW* and ABI sequenced. All PCR reactions for genomic DNA amplification were performed using Phanta Max Super-Fidelity DNA polymerase (Vazyme, Piscataway, NJ). The primers used are listed in Table S1. Sequence assembly and alignment was performed by CLC Sequence Viewer software (www.qiagenbioinformatics.com).

### DNA extraction and genotyping

Genomic DNA of fresh leaves was extracted using the CTAB method (Saghai-Maroof *et al.* 1984). Genomic DNA of seeds was isolated using a previously described method with some modifications (Guan *et al.* 2012). Briefly, 0.02–0.05 g endosperms were cut from the apex of the seed, and were collected into the well of PCR plates. In each wall, 50 μl 0.1 mol/L NaOH was added, followed by boiling for 10 min at 100° in a thermal cycler. Then 50 μl of TE (10 mM Tris-HCl, 1 mM EDTA, pH 2.0) buffer was added to each well, and the mixture was centrifuged at 2500 rpm for 1 min at room temperature. The supernatant was used as the template for PCR amplification. PCR products were subjected to electrophoresis on a 6% polyacrylamide gel, and then silver stained for visualization.

### RNA extraction and quantitative reverse transcription PCR (qRT-PCR)

Leaves from four uniform seedlings that were planted in a plastic pot (5 cm in diameter and 6 cm deep) containing nutrient soil were pooled for RNA extraction using Trizol reagent (ThermoFisher Scientific, Waltham, MA), and the gDNA wiper Mix (Vazyme) was used to remove DNA. HiScript II qRT SuperMix II (Vazyme) was used to synthesize complementary DNA (cDNA). qRT-PCR was performed using the SYBR Green RT-PCR kit (ThermoFisher Scientific). The normalized expression levels were analyzed as described in Livak and Schmittgen (2001). Three biological replications were performed for *muw* and wild-type samples respectively. The gene-specific primers used are listed in Table S1.

### Data availability

The authors state that all data necessary for confirming the conclusions presented in this article are represented fully within the article and Supplemental Material. Additionally, File S1 contains the 11,435-bp sequence flanking the deletion breakpoint (this sequence was also deposited with GenBank under accession No. MH675928). Plant materials in this study are available upon request. Supplemental material available at Figshare:https://doi.org/10.25386/genetics.7273028

## Results

### The *muw* mutant shows pleiotropic abnormalities in agronomic, cytological, and physiological traits

**Figure 1:**
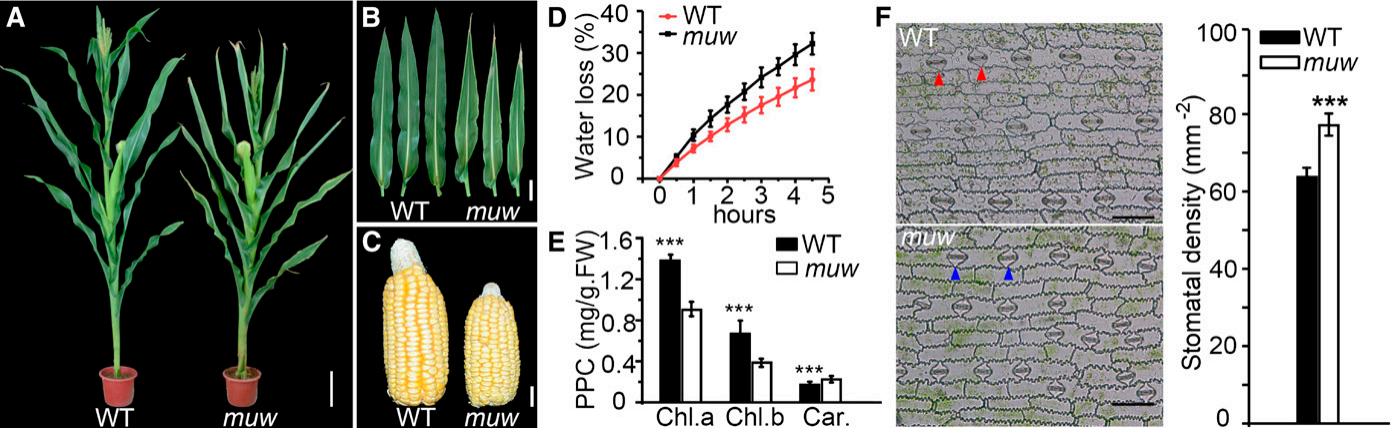
Phenotypic comparison between the *muw* mutant and wild type. (A) Overall appearance of the wild type and the *muw* mutant plants after pollination. Bar, 20 cm. (B) Leaves from wild-type and *muw* plants after pollination. Bar, 10 cm. (C) Ears of the *muw* mutant and wild type. Bar, 2 cm. (D) Leaf water loss of the wild type and the *muw* mutant at different time points. Values are the mean ± SE, *n* = 3, 12 leaves per replicate. (E) Chlorophyll a (Chl.a, mg/g FW), chlorophyll b (Chl.b, mg/g FW), and carotenoid (Car., mg/g FW) concentration in wild-type and *muw* leaves at the seeding stage. PPC, photosynthetic pigment concentration. Data are shown as the mean ± SE, *n* = 12 individuals (*** *P* < 0.001, Student’s *t*-test). (F) Stomatal density in the lower epidermis of leaves of the wild type and the *muw* mutant under optical microscopy. Red and blue triangles indicate the stomata in the wild type and *muw* mutant, respectively. Stomatal density was detected in 20 individuals and is shown as the mean ± SE (*** *P* < 0.001, Student’s *t*-test). Bar, 100 µm.

Phenotypic comparisons showed that the overall appearance of the *muw* mutant was highly similar to that of the wild-type individual (Figure 1A and Table 1), but the *muw* mutant was ∼13 cm shorter than the wild type and exhibited dehydrated leaf tips beginning at the five-leaf seedling stage. The leaf tip dehydration phenotype was more serious with growth and development of the individual, resulting in irreversible damage to leaf tips and margins (Figure 1B). We also evaluated agronomically important traits carefully and found that the *muw* mutant produced a smaller ear relative to wild type. And EL, ED, and CD of the *muw* were significantly decreased relative to those of the wild type (by 16.1, 14.2, and 10.1%, respectively) (Figure 1C and Table 1). Moreover, compared to the wild type, KL, KW, and HKW of the *muw* were reduced by 20.9, 12.6, and 21.0%, respectively, which resulted in a smaller kernel size and a serious loss of grain yield in the *muw* (Table 1). However, the KRN and KT of the *muw* mutant did not differ significantly from that of the wild type (Table 1). Because excessive water loss usually leads to wilting in plants (Chen *et al.* 2004), we measured the water-loss rate and found 35.6–43.9% faster water loss in the *muw* leaves than in wild-type leaves (Figure 1D). Additionally, in comparison with the wild type, Chl. a and Chl. b concentrations in the *muw* leaves were 25.3 and 25.7% lower, respectively, but Car. concentration was 22.0% higher (Figure 1E), and the decreased Chl. a and b led to light-green leaves in the *muw* plants.

**Table 1:** Phenotypic comparison between the *muw* mutant and the wild type

Trait	Wild type (WT) mean ± SD *^a^*	Mutant (*muw*) mean ± SD*^a^*	*P*-value*^b^*
Kernel thickness (mm)	9.94 ± 0.27	9.74 ± 0.21	0.07
Kernel length (mm)	21.32 ± 0.27	16.86 ± 0.33	1.45E−17
Kernel width (mm)	19.04 ± 0.36	16.64 ± 0.25	1.73E−13
100-kernel weight (g)	29.52 ± 0.59	23.33 ± 0.41	1.37E−31
Ear diameter (mm)	50.90 ± 1.59	43.68 ± 1.89	4.90E−06
Cob diameter (mm)	29.55 ± 1.20	26.56 ± 1.40	1.41E−08
Ear length (mm)	138.35 ± 2.33	116.05 ± 1.91	3.19E−29
Kernel row number	16.2 ± 1.01	16.1 ± 0.99	0.77
Plant height (cm)	165.05 ± 1.69	152.05 ± 2.91	3.12E−19
Ear height (cm)	67.70 ± 1.10	62.50 ± 1.32	9.60E−16

a The mean values ± SD (SD, calculated from the variation observed over plants).

b *P*-values estimated by Student’s *t*-test. All traits were phenotyped in 60 individuals.

To understand the possible reasons for the increased water loss in the *muw* mutant, we characterized stomatal density, cuticle permeability, ABA concentration, ABA response, vascular bundle integrity, and water conductivity because these characteristics lead to wilting in plants (Sagi *et al.* 1999; Koizumi *et al.* 2007; Kim *et al.* 2014; Lee and Suh 2015; Y. Liu *et al.* 2015). Our results showed that the stomatal density of the *muw* leaves was 90 mm^−2^, which was 30% greater than that in the wild type (63 mm^−2^) (Figure 1F). However, the *muw* leaves had a normal cuticle layer and showed a similar chlorophyll efflux rate to that of the wild type (Figure S1A). In addition, differences in the ABA concentration between the *muw* mutant and the wild type were also not significant (Figure S1B), suggesting that the capacity to accumulate ABA under drought stress was not impaired in the *muw* mutant. Germination of the *muw* and wild-type seeds in response to exogenous ABA was also analyzed. Relative to control (seeds soaked with water), germination percentage of the *muw* seeds treated with exogenous ABA was decreased 10%, and germination of seeds was delayed. A similar phenomenon was also observed in wild-type seeds (Figure S1C). These results indicated that *muw* mutants exhibit a normal ABA response. Our analyses also showed that the number of vascular bundles (168.0 for the *mu*w and 167.5 for WT) and vessels from the culm (2.18 for the *muw* and 2.16 for WT) were similar in both genotypes (Figure S1, D and E), which indicated that there were no significant differences in the ability to transport water between the *muw* mutant and the wild type.

**Figure 2:**
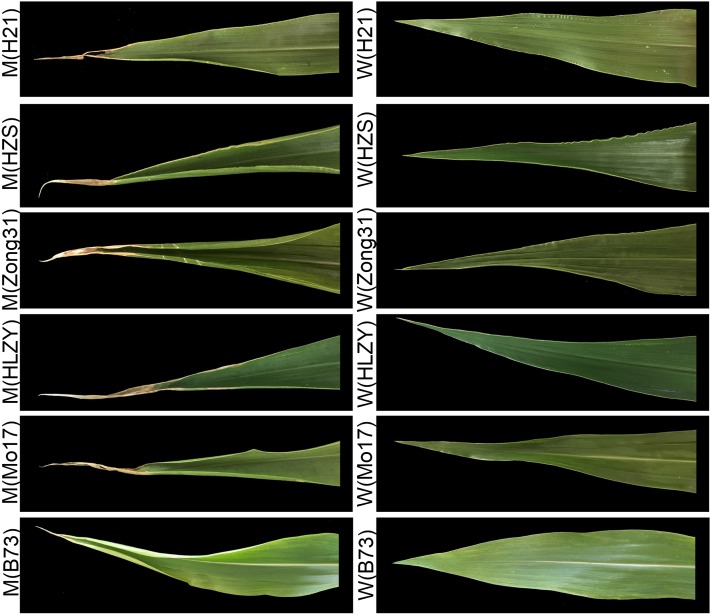
The phenotype of the leaves in F_2_ populations derived from six different F_1_ combinations (B73×*muw*, Mo17×*muw*, HLZY×*muw*, Zong31×*muw*, HZS×*muw*, H21×*muw*). Upper panel shows leaves of F_2_ plants that exhibited *muw* mutant phenotype in six F_2_ populations; lower panel shows the corresponding wild type.

Furthermore, to detect the effects of the *muw* mutation in different genetic backgrounds, we crossed the *muw* mutant to six maize inbred lines. All F_1_ plants of the six combinations exhibited wild-type phenotypes, and the F_2_ plants of each combination exhibited phenotypic segregation in agreement with a ratio of 3 wild type: 1 mutant producing wilted leaves (Figure 2 and Table S4), showing that the *muw* allele results in a similar effect in different genetic backgrounds, and is inherited as a single recessive locus.

### A megabase-scale deletion is responsible for the multi-trait abnormalities in the *muw* mutant

**Figure 3:**
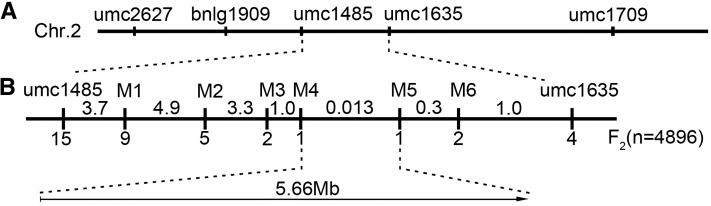
Map-based cloning of the *muw* mutation. (A) The *muw* mutation was mapped to the umc1485 and umc1635 interval on chromosome 2 by bulked-segregant analysis. (B) The *muw* mutation was fine-mapped to a 5.66-Mb region flanked by M4 and M5. The numbers below and above the horizontal line, respectively, represent recombinational events and genetic distance (cM).

To isolate the *muw*, we performed bulked-segregant analysis and mapped the *muw* mutation to the marker interval flanked by the *umc1485* and *umc1635* markers on chromosome 2 (Figure 3A). For fine mapping, we identified 38 recombinants by screening 4896 F_2_ plants using these two flanking markers. Additionally, we developed six SSR markers (Table S1) that showed polymorphisms between the two parents to further screen the recombinants, and two novel chromosome events were detected within the *M4*–*M5* interval, a region of about 5.66-Mb in the B73 reference genome V4 (Figure 3B). Through further screening of 9600 F_2_ individuals derived from the *muw* × V54 cross, we found that the genetic distance between *M4* and *M5* was 0.013 cM.

Owing to the low recombination rate between *M4* and *M5*, we reconstructed the genetic map of the target interval using 960 F_2_ individuals derived from the cross of Lian87 (a wild-type, near-isogenic line of the *muw* mutant but harboring *MUW* allele) × V54, and found that the genetic distance between *M4* and *M5* was 0.73 cM, which is notably greater than the 0.013 cM distance discussed above, and strongly supports the 5.66-Mb physical distance in the B73 reference genome. Therefore, we hypothesized that a large chromosomal structural variation occurred in the *M4*–*M5* region in the *muw* mutant. Furthermore, we used 100 pairs of newly designed InDel markers in this region to screen for the genetic diversity between the wild type and the *muw* mutant, and found that 96 markers could amplify products in the wild type but not in the *muw* mutant, indicating that a large DNA segment in the 5.66-Mb region might be deleted in the *muw* mutant. We also hypothesized that this megabase-scale deletion is responsible for the abnormal phenotypes in the *muw* mutant.

### Identification and verification of the deleted genomic region

**Figure 4:**
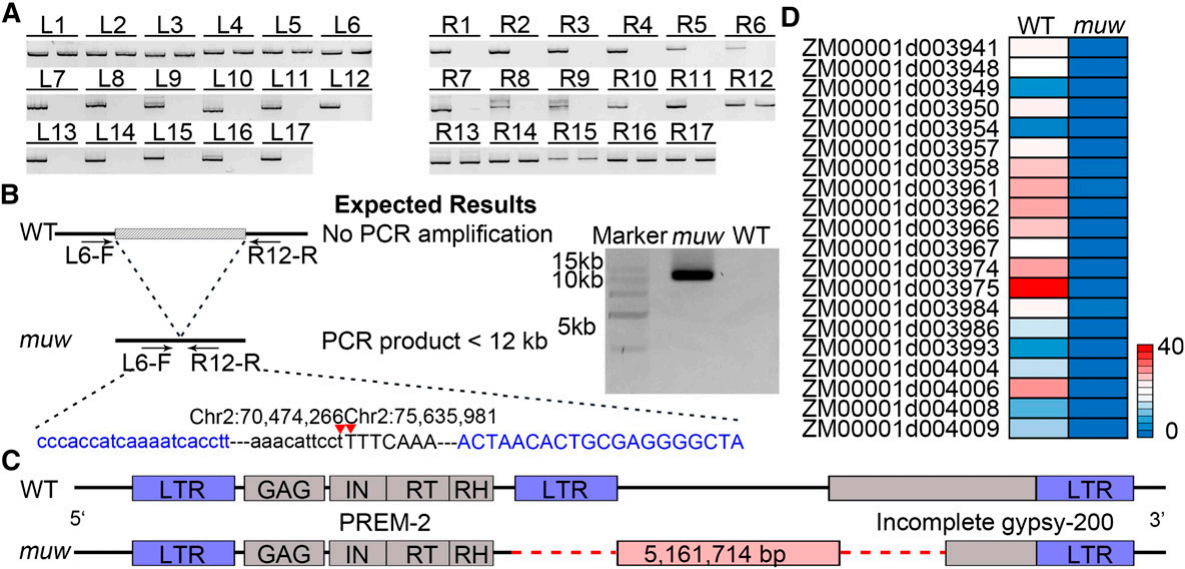
The 5.16-Mb deletion explains the abnormal phenotypes of the *muw* mutant. (A) Analysis of the position of the deleted fragment. L1–L17 and R1–R17 primer pairs were used to amplify this genomic region in the wild type (left) and the *muw* mutant (right). (B) Validation of the predicted deletion by PCR and sequencing. The arrows represent primer binding sites. The lower portion of the diagram shows the partial sequence of the PCR product spanning the deletion junction site in the *muw*. The 5′-region flanking the deletion junction site in the *muw* is shown in lower-case letters, while the 3′-region is in upper-case letters. The blue lower-case letters and upper-case letters represent binding sequences of L6-F and R12-R primers, respectively. The breakpoints in the *MUW* are indicated by two red triangles. (C) Schematic comparison of the wild type and the *muw* mutant sequences flanking the left and right breakpoints of the deletion. The breakpoints of the deletion fall within a PREM-2 retrotransposon and an incomplete gypsy LTR-retrotransposon. The PREM-2 retrotransposon contains the 1321-bp 5′-LTR and the full-length open reading frames which encode the gag (GAG), an integrase (IN), a reverse transcriptase (RT), and a RNase H (RH). The red dashed line represents the deleted 5.16-Mb sequence in *muw*. The sequences near the deletion breakpoints in the *muw* mutant share low sequence similarity. (D) The relative expression of 20 genes within the deleted segment in leaf tissues of the wild-type (WT) and *muw* seedlings. All 20 genes were expressed in leaf tissues of wild type but not in the *muw* mutant.

To determine the breakpoints at both ends of *MUW*, we designed 17 markers (*L1*–*L17*, referred to as left-end markers) near *M4*, as well as 17 markers (*R1*–*R17*, referred to as right-end markers) near *M5*, and used these markers for screening the genetic polymorphism between the wild type and the *muw* mutant. If a pair of PCR primers can amplify the expected products in the wild type but not in the *muw* mutant, this indicates that the DNA segment may be absent in the *muw* mutant. Markers *L1*–*L6* amplified the same sized products in the wild type and the *muw* mutant, whereas the other markers only amplified products in the wild type, indicating that the left breakpoint might be located between *L6* (chr2: 70,464,146 bp) and *L7* (chr2: 70,475,791 bp) (Figure 4A). Similarly, the right breakpoint might be located between *R11* (chr2: 75,635,510 bp) and *R12* (chr2: 75,636,014 bp) (Figure 4A). Therefore, we believe the deleted segment occurred between *L6* and *R12*. To confirm these findings, we carried out a genomic PCR assay using primers *L6-F* and *R12-R*; a PCR product of ∼12-kb was amplified in the *muw* mutant. However, the segment flanked by the *L6* and *R12* is too long to amplify product in the wild type (Figure 4B). After sequencing the PCR product, an 11,435-bp sequence was obtained and deposited in the NCBI database (File S1, Accession No. MH675928). A 10,757-bp long segment (1–10,757-bp from 5′- to 3′-end) of the 11,435-bp sequence was well matched with the sequence from 70,463,510 to 70,474,266-bp on chromosome 2 of B73 RefGen V4, and a 678-bp sequence (from 10,758 to 11,435-bp) was mapped to the segment from 75,635,981 to 75,636,658-bp on chromosome 2. Sequence alignment revealed that a 5,161,714-bp (from 70,474,267 to 75,635,980-bp on chromosome 2 of B73 RefGen V4) sequence is entirely absent from the *muw* mutant’s genome. Thus, the left and right breakpoints for the deletion occurred between 70,474,266 and 70,474,267-bp and between 75,635,980 and 75,635,981-bp (Figure 4B) on chromosome 2 of the wild-type genome, respectively, based on the B73 RefGen V4. Within the deleted 5.16-Mb sequence, 48 genes are annotated in the B73 reference genome V4 (Table S2).

We then aligned the 11,435-bp sequence of wild-type allele *muw* immediately flanking the breakpoint against a reference collection of repeats using CENSOR software (Kohany *et al.* 2006), and found that, in the *MUW* allele, the flanking sequence of left breakpoint encodes a Ty1-copia-like maize retrotransposon, PREM-2 (pollen retroelement maize-2) (Turcich *et al.* 1996), which is bounded by two 1321-bp LTRs and contains coding domains of GAG (group-specific antigen), integrase (IN), reverse transcriptase (RT), and RNase H (RH) (Figure 4C). The LTR at the 3′-end of RH is absent in the *muw* allele. The left breakpoint in the *MUW* allele occurs between the 3′-end of RH and the neighboring LTR (Figure 4C). The flanking sequence of the right breakpoint harbors an incomplete gypsy-200 TE in the *MUW* allele, and the right breakpoint occurs in the internal portion of the gypsy-200 (Figure 4C). Thus, the *muw* mutant deleted the entire sequence between the PREM-2 and the incomplete gypsy-200 and parts of the PREM-2 and incomplete gypsy-200. Additionally, we retrieved a 1-kb DNA sequence on each side of each breakpoint from the *MUW* allele (File S2), and the 4-kb sequence (2-kb for each breakpoint) was used to conduct pairwise alignment and analysis of the percentage of adenine/thymine. The result showed only 41.3% sequence similarity, suggesting low sequence homology near the breakpoints. Moreover, the percentage of A/T is 69% in the upstream 200-bp region of the left breakpoint and 63% in the downstream 200-bp region of the right breakpoint, showing that the A/T content at breakpoint regions is more abundant than in regions 1-kb away from the breakpoints (Figure S2), and is also more abundant than the average A/T content (53.1%) of the maize genome (Jiao *et al.* 2017).

### Transcripts for 20 genes in the deleted region are not detectable in the *muw* mutant

Expression data for these 48 genes retrieved from the qteller database (www.qteller.com) showed that all 48 genes are spatiotemporally expressed in different tissues and organs, or under abiotic conditions, indicating that they function in specific biological processes, cellular components, or stress responses. Among them, 20 genes that are expressed in leaf tissue were selected to measure their expression level by qRT-PCR. We found that these 20 genes were expressed in Lian87 leaves, but none was expressed in the *muw* mutant (Figure 4D). The gene expression results further exemplified the loss of these genes in the *muw* mutant. Furthermore, we attempted to amplify all 48 genes in Lian87, the *muw* mutant, and 60 other maize inbred lines using gene-specific primers. All of the 48 genes were amplified in Lian87 and in the 60 inbred lines, but not in the *muw* mutant (Table S3).

The abnormal phenotypes of the *muw* mutant may be associated with the loss of these genes. *ZM00001d003980*, which encodes a subtilisin-like serine proteinase, is an ortholog of *Arabidopsis stomatal density and distribution1* (*SDD1*). *Arabidopsis sdd1* mutants exhibit a twofold to fourfold increase of stomatal density and enhanced water loss (Berger and Altmann 2000; Vráblova *et al.* 2017). Overexpressing *ZmSDD1* in maize decreases this water loss by reducing stomatal density (Y. Liu *et al.* 2015). Additionally, subtilisin-like serine protease-encoding genes are also specifically expressed in early developing kernels (López *et al.* 2017). Therefore, we suggest that *ZM00001d003980* might be involved in stomatal and kernel development. *ZM00001d003986* is a member of the leucine-rich repeat (LRR) receptor-like kinase superfamily. Some members of this superfamily, such as CLAVATA1 (CLV1), CLAVATA1-related BARELY ANY MERISTEM1 (BAM1), BAM2, and BAM3, are key regulators for cell proliferation and stem cell maintenance in dicots and monocots (DeYoung *et al.* 2006; DeYoung and Clark 2008; De Smet *et al.* 2009; Somssich *et al.* 2016). Another two deleted genes, *ZM00001d003948* and *ZM00001d003981*, encode a putative DnaJ heat-shock protein and a DnaJ chaperone, respectively. DnaJ chaperones are involved in heat-stress tolerance in maize (Young *et al.* 2001; Sun *et al.* 2012; Klein *et al.* 2014; Hu *et al.* 2015). Despite a lack of direct evidence, we assume that the numerous phenotypic abnormities of the *muw* mutant are the combined effect of the loss of these genes encoded in the 5.16-Mb deleted region.

## Discussion

In maize, plant height is highly related to internode development, which is determined by differentiation of the shoot apical meristem (Teng *et al.* 2013; Leiboff *et al.* 2015; Xing *et al.* 2015), and internode length, which is determined by the activity of the intercalary meristem (Hattori *et al.* 2009; Knöller *et al.* 2010). Plant stomata are derived from asymmetrical division of protodermal cells on the outermost tissue layer of the shoot apical meristem and symmetrical division of the guard mother cell (Bergmann *et al.* 2004). Kernel number and size are determined by inflorescence and floral development (Huang *et al.* 2009; Miura *et al.* 2010; Bai *et al.* 2016), cell division of the episperm during floral development, and the influx of carbohydrates after pollination (Song *et al.* 2007; Li *et al.* 2011; Zhang *et al.* 2012; Ishimaru *et al.* 2013). These biological processes are considered to be developmentally interrelated, and are controlled by different regulatory pathways. Here, we report a maize mutant with multiple abnormal phenotypes involving plant height, seed number and size, leaf stoma number, and other physiological characteristics, and is referred to as the *multi-trait weakened* (*muw*) mutant. Map-based cloning delimited the *muw* mutation into a 5.16-Mb deleted genomic segment, which is responsible for these defective phenotypes in the *muw* mutant.

### Deletion of a large DNA segment in the maize genome causes a wide range of phenotypic defects

Genomic deletion is a universal genetic phenomenon. Cytogenetic studies have shown that the deficiency in pale-yellow and white seedling mutants is associated with losses of terminal segments of the short arm of chromosome 9 (McClintock 1944). With the advance of next-generation sequencing, comparative genomics has revealed a massive amount of large-scale genomic deletions among maize genomes, which have played important roles in evolution and functional impacts (Saxena *et al.* 2014; Zhang *et al.* 2018). For example, the 147-kb deletion within *qHSR1* from HZ4 contains *ZmWAK*, causing susceptibility to head smut in maize, and appears to have occurred after domestication and spread among maize germplasm (Zuo *et al.* 2015). Previous studies showed that loss of function of key genes leads to defective phenotypes involving multiple phytomers, and deletion eliminates gene function. In addition to deletion of key genes, several large deletions have altered a wide range of biological functions. The 1.2-Mb deletion induced by γ-irradiation within the *opaque2* deletion line 107 resulted in loss of the 27- and 50-kD γ-zein-encoding genes and nine other genes on chromosome 7 (Yuan *et al.* 2014). In the mutant 1115, developed from B73 by γ-irradiation, a large deletion (∼1.7 Mb) was identified on chromosome 3 by exome-seq and bulked segregant RNA-seq (BSR-seq), which covers 26 genes and leads to small and opaque kernels (Jia *et al.* 2016). In this study, the deletion of a 5.16-Mb segment on chromosome 2 in the *muw* mutant led to a loss of 48 genes, which was responsible for a set of phenotypic defects, including wilting leaves, poor yield performance, reduced plant height, rapid water loss, and increased stomatal density.

### Functional redundancy buffers the detrimental effects caused by deletion

Some large-segment deletions, such as the 5.16-Mb deletion found in the *muw* mutant, do not cause severe defective phenotypes or lethality, which may be partially explained by the functional unimportance of a deleted gene and/or redundancy with other genes. First, megabase-scale presence/absence variations (PAVs) are frequently found when comparing the genomes of two diverse inbred lines. For example, 200 and 126 PAV sequences that were >5 kb were detected in B73 and Mo17, respectively. Among of these PAVs, a longest 2.9-Mb genomic segment harboring 66 annotated genes is present on chromosome 6 in B73 but is absent in Mo17 (Sun *et al.* 2018); however, Mo17 does not show any serious defective or lethal phenotypes relative to B73, despite the abundant quantitative variation between B73 and Mo17 in a wide range of phenotypic and physiological traits. Second, the functions of the missing genes can be fully or partially compensated by their paralogs. A widely accepted view is that maize, as a paleopolyploid, underwent a recent whole-genome duplication followed by genomic diploidization involving complex chromosomal events (Bennetzen 2007; Wei *et al.* 2007). Although genes are rapidly silenced and/or lost from the duplicated genome, functional redundancy exists widely in the maize genome. For example, the duplicate *SQUAMOSA promoter binding protein* (SBP)-box transcription factors encoded by *unbranched2* (*ub2*) and *unbranched3* (*ub3*), function together redundantly to regulate KRN in maize (Chuck *et al.* 2014; L. Liu *et al.* 2015). Although the *UB3-mum4* and *UB2-mum3* single mutants do not show an obvious change in KRN, double mutants of *UB3-mum4* and *UB2-mum3* show a significant decrease in KRN relative to the wild type (Chuck *et al.* 2014). ALTERNATIVE DISCORDIA1 (ADD1) is 96% identical in amino acid sequence to DISCORDIA1 (DCD1), which controls preprophase band formation in asymmetrically dividing cells in maize (Wright *et al.* 2009). Mutants of *dcd1* have specifically misoriented asymmetric cell divisions in the leaf epidermis. Loss of function of *add1* does not produce a noticeable phenotype, while knock down of *add1* (RNAi) and *dcd1* (RNAi) causes misorientation of symmetric and asymmetric cell divisions (Wright *et al.* 2009). Thus, the functional loss of a gene can be complemented by its closely related paralogs. In the B73 reference genome V4, the 48 genes lost in the *muw* mutant have a total of 460 paralogs, averaging 9.58 paralogs per gene. For example, a total of 45 putative subtilisin-like serine proteases are encoded in the B73 genome, and one, or several, of them might provide functional complementation to loss of Z*M00001d003980* (Jiao *et al.* 2017). In addition, a total of 188 DnaJ proteins have been annotated in the B73 genome (Jiao *et al.* 2017). These putative paralogs may completely or partially complement the functions of the 48 deleted genes. Thus, functional redundancy of genes might partially explain the absence of severely detrimental or lethal phenotypes of the *muw* mutant.

### Putative mechanism that caused the megabase-scale deletion

DNA double-strand breaks are the most harmful form of DNA damage. Large-scale deletions can occur during the repair of double-strand breaks by two main pathways: NAHR and NHEJ (Chen *et al.* 2010). The mechanism of genomic deletions may be associated with the DNA sequences at the downstream and upstream of the deletion breakpoints, which may help us to understand the mechanisms for creation of chromosomal deletions (Abeysinghe *et al.* 2003; Gu *et al.* 2008; Jennes *et al.* 2011). NAHR is a type of genetic recombination that requires the presence of homologous DNA sequences (≥200 bp) to act as a template to directly repair the complementary strand (Jennes *et al.* 2011; Yang *et al.* 2016). For example, recombination between the internal regions of two barley retroelement (BARE) copies generates a recombinant BARE and a shortened copy with a 28-kb deletion in barley (*Hordeum vulgare*) (Shang *et al.* 2017); intrachromosomal recombination between two LTRs leads to a solo LTR in rice (Vitte and Panaud 2003). However, the lack of homologous or microhomologous sequences near the deletion breakpoints in the *muw* and the wild-type alleles makes it highly unlikely that homology-based mechanisms are involved. In other words, NAHR might not be a key mechanism causing the 5.16-Mb sequence deletion in the *muw* mutant. In contrast, NHEJ refers to the repair of double-strand breaks in which the broken ends are directly ligated (Yang *et al.* 2016). NHEJ is a major pathway for the repair of double-strand breaks in plant and mammalian cells (Gorbunova and Levy 1997; Lieber 2010), and very large deletions are thought to be generated by NHEJ (Sachs *et al.* 2000). In *Arabidopsis*, NHEJ repair of damaged DNA is the main mechanism involved in the formation of deletions (Britt 1996). Thus, NHEJ is likely responsible for the deletion in the *muw* mutant. Advances in the mechanism of genomic deletion depend mainly on research with human subjects (Chen *et al.* 2010; Jennes *et al.* 2011; Ottaviani *et al.* 2014). There are putatively three enzymatic activities required for repair of double-strand DNA breaks by the NHEJ pathway: (a) nucleases to remove damaged DNA, (b) polymerases to aid in the repair, and (c) a ligase to restore the phosphodiester backbone (Chen *et al.* 2010) (Figure S3). The mechanism of the 5.16-Mb deletion in the *muw* mutant still needs to be further studied. Additionally, adenine/thymine-enriched sequences are thought to play a role in the appearance of large deletions (Abeysinghe *et al.* 2003; Jennes *et al.* 2011; Enggaard Hoeffding *et al.* 2014). The proportion of adenine/thymine in the flanking sequences of the breakpoint in the *muw* mutant is higher than the proportion of the whole genome, which may also be involved in the genomic deletion in this mutant.

## References

[bib1] Abeysinghe S. S. Chuzhanova N. Krawczak M. Ball E. V. Cooper D. N., 2003 Translocation and gross deletion breakpoints in human inherited disease and cancer I: nucleotide composition and recombination-associated motifs. Hum. Mutat. 22: 229–244. 10.1002/humu.10254 12938088

[bib2] Arnon D. I., 1949 Copper enzymes in isolated chloroplasts. Polyphenoloxidases in beta vulgaris. Plant Physiol. 24: 1–5. 16654194 10.1104/pp.24.1.1 PMC437905

[bib3] Bai X. Huang Y. Mao D. Wen M. Zhang L., 2016 Regulatory role of FZP in the determination of panicle branching and spikelet formation in rice. Sci. Rep. 6: 19022 10.1038/srep19022 26744119 PMC4705600

[bib4] Bennetzen J. L., 2007 Patterns in grass genome evolution. Curr. Opin. Plant Biol. 10: 176–181. 10.1016/j.pbi.2007.01.010 17291821

[bib5] Berger D. Altmann T., 2000 A subtilisin-like serine protease involved in the regulation of stomatal density and distribution in *Arabidopsis thaliana*. Genes Dev. 14: 1119–1131. 10809670 PMC316574

[bib6] Bergmann D. C. Lukowitz W. Somerville C. R., 2004 Stomatal development and pattern controlled by a MAPKK kinase. Science 304: 1494–1497. 10.1126/science.1096014 15178800

[bib7] Britt A. B., 1996 DNA-damage and repair in plants. Annu. Rev. Plant Physiol. Plant Mol. Biol. 47: 75–100. 10.1146/annurev.arplant.47.1.75 15012283

[bib8] Brugière N. Zhang W. Xu Q. Scolaro E. J. Lu C., 2017 Overexpression of RING domain E3 ligase ZmXerico1 confers drought tolerance through regulation of ABA homeostasis. Plant Physiol. 175: 1350–1369. 10.1104/pp.17.01072 28899960 PMC5664481

[bib9] Brunner S. Fengler K. Morgante M. Tingey S. Rafalski A., 2005 Evolution of DNA sequence nonhomologies among maize inbreds. Plant Cell 17: 343–360. 10.1105/tpc.104.025627 15659640 PMC548811

[bib10] Chen G. Sagi M. Weining S. Krugman T. Fahima T., 2004 Wild barley *eibi1* mutation identifies a gene essential for leaf water conservation. Planta 219: 684–693. 10.1007/s00425-004-1277-7 15197591

[bib11] Chen J. M. Cooper D. N. Ferec C. Kehrer-Sawatzki H. Patrinos G. P., 2010 Genomic rearrangements in inherited disease and cancer. Semin. Cancer Biol. 20: 222–233. 10.1016/j.semcancer.2010.05.007 20541013

[bib12] Chia J. M. Song C. Bradbury P. J. Costich D. de Leon N., 2012 Maize HapMap2 identifies extant variation from a genome in flux. Nat. Genet. 44: 803–807. 10.1038/ng.2313 22660545

[bib13] Chuck G. S. Brown P. J. Meeley R. Hake S., 2014 Maize SBP-box transcription factors *unbranched2* and *unbranched3* affect yield traits by regulating the rate of lateral primordia initiation. Proc. Natl. Acad. Sci. USA 111: 18775–18780. 10.1073/pnas.1407401112 25512525 PMC4284592

[bib14] De Smet I. Voss U. Jurgens G. Beeckman T., 2009 Receptor-like kinases shape the plant. Nat. Cell Biol. 11: 1166–1173. 10.1038/ncb1009-1166 19794500

[bib15] DeYoung B. J. Clark S. E., 2008 BAM receptors regulate stem cell specification and organ development through complex interactions with CLAVATA signaling. Genetics 180: 895–904. 10.1534/genetics.108.091108 18780746 PMC2567389

[bib16] DeYoung B. J. Bickle K. L. Schrage K. J. Muskett P. Patel K., 2006 The CLAVATA1-related BAM1, BAM2 and BAM3 receptor kinase-like proteins are required for meristem function in *Arabidopsis*. Plant J. 45: 1–16. 10.1111/j.1365-313X.2005.02592.x 16367950

[bib17] Enggaard Hoeffding L. K. Hansen T. Ingason A. Doung L. Thygesen J. H., 2014 Sequence analysis of 17 NRXN1 deletions. Am. J. Med. Genet. B. Neuropsychiatr. Genet. 165B: 52–61. 24339137 10.1002/ajmg.b.32204

[bib18] Feuk L. Carson A. R. Scherer S. W., 2006 Structural variation in the human genome. Nat. Rev. Genet. 7: 85–97. 10.1038/nrg1767 16418744

[bib19] Fu H. Dooner H. K., 2002 Intraspecific violation of genetic colinearity and its implications in maize. Proc. Natl. Acad. Sci. USA 99: 9573–9578. 10.1073/pnas.132259199 12060715 PMC123182

[bib20] Goettel W. Ramirez M. Upchurch R. G. An Y. Q., 2016 Identification and characterization of large DNA deletions affecting oil quality traits in soybean seeds through transcriptome sequencing analysis. Theor. Appl. Genet. 129: 1577–1593. 10.1007/s00122-016-2725-z 27179525 PMC4943983

[bib21] Gorbunova V. Levy A. A., 1997 Non-homologous DNA end joining in plant cells is associated with deletions and filler DNA insertions. Nucleic Acids Res. 25: 4650–4657. 10.1093/nar/25.22.4650 9358178 PMC147090

[bib22] Gu W. Zhang F. Lupski J. R., 2008 Mechanisms for human genomic rearrangements. PathoGenetics 1: 4 10.1186/1755-8417-1-4 19014668 PMC2583991

[bib23] Guan H. Liu C. Zhao Y. Zeng B. Zhao H., 2012 Characterization, fine mapping and expression profiling of *Ragged leaves1* in maize. Theor. Appl. Genet. 125: 1125–1135. 10.1007/s00122-012-1899-2 22648613

[bib24] Hastings P. J. Lupski J. R. Rosenberg S. M. Ira G., 2009 Mechanisms of change in gene copy number. Nat. Rev. Genet. 10: 551–564. 10.1038/nrg2593 19597530 PMC2864001

[bib25] Hattori Y. Nagai K. Furukawa S. Song X. J. Kawano R., 2009 The ethylene response factors SNORKEL1 and SNORKEL2 allow rice to adapt to deep water. Nature 460: 1026–1030. 10.1038/nature08258 19693083

[bib26] Hu X. Yang Y. Gong F. Zhang D. Zhang L., 2015 Protein sHSP26 improves chloroplast performance under heat stress by interacting with specific chloroplast proteins in maize (*Zea mays*). J. Proteomics 115: 81–92. 10.1016/j.jprot.2014.12.009 25540934

[bib27] Huang X. Qian Q. Liu Z. Sun H. He S., 2009 Natural variation at the *DEP1* locus enhances grain yield in rice. Nat. Genet. 41: 494–497. 10.1038/ng.352 19305410

[bib28] Hurles M. E. Dermitzakis E. T. Tyler-Smith C., 2008 The functional impact of structural variation in humans. Trends Genet. 24: 238–245. 10.1016/j.tig.2008.03.001 18378036 PMC2869026

[bib29] Ishimaru K. Hirotsu N. Madoka Y. Murakami N. Hara N., 2013 Loss of function of the IAA-glucose hydrolase gene *TGW6* enhances rice grain weight and increases yield. Nat. Genet. 45: 707–711. 10.1038/ng.2612 23583977

[bib30] Jennes I. de Jong D. Mees K. Hogendoorn P. C. Szuhai K., 2011 Breakpoint characterization of large deletions in *EXT1* or *EXT2* in 10 multiple osteochondromas families. BMC Med. Genet. 12: 85 10.1186/1471-2350-12-85 21703028 PMC3152881

[bib31] Jia S. Li A. Morton K. Avoles-Kianian P. Kianian S. F., 2016 A population of deletion mutants and an integrated mapping and Exome-seq pipeline for gene discovery in maize. G3 (Bethesda) 6: 2385–2395. 10.1534/g3.116.030528 27261000 PMC4978893

[bib32] Jiao Y. Peluso P. Shi J. Liang T. Stitzer M. C., 2017 Improved maize reference genome with single-molecule technologies. Nature 546: 524–527. 28605751 10.1038/nature22971 PMC7052699

[bib33] Kidd J. M. Graves T. Newman T. L. Fulton R. Hayden H. S., 2010 A human genome structural variation sequencing resource reveals insights into mutational mechanisms. Cell 143: 837–847. 10.1016/j.cell.2010.10.027 21111241 PMC3026629

[bib34] Kim H. K. Park J. Hwang I., 2014 Investigating water transport through the xylem network in vascular plants. J. Exp. Bot. 65: 1895–1904. 10.1093/jxb/eru075 24609652

[bib35] Klein R. D. Chidawanyika T. Tims H. S. Meulia T. Bouchard R. A., 2014 Chaperone function of two small heat shock proteins from maize. Plant Sci. 221–222: 48–58. 10.1016/j.plantsci.2014.01.012 24656335

[bib36] Knöller A. S. Blakeslee J. J. Richards E. L. Peer W. A. Murphy A. S., 2010 *Brachytic2/ZmABCB1* functions in IAA export from intercalary meristems. J. Exp. Bot. 61: 3689–3696. 10.1093/jxb/erq180 20581123 PMC2921204

[bib37] Kohany O. Gentles A. J. Hankus L. Jurka J., 2006 Annotation, submission and screening of repetitive elements in Repbase: RepbaseSubmitter and Censor. BMC Bioinformatics 7: 474 10.1186/1471-2105-7-474 17064419 PMC1634758

[bib38] Koizumi K. Ookawa T. Satoh H. Hirasawa T., 2007 A wilty mutant of rice has impaired hydraulic conductance. Plant Cell Physiol. 48: 1219–1228. 10.1093/pcp/pcm092 17634180

[bib39] Lai J. Li R. Xu X. Jin W. Xu M., 2010 Genome-wide patterns of genetic variation among elite maize inbred lines. Nat. Genet. 42: 1027–1030. 10.1038/ng.684 20972441

[bib40] Lander E. S. Green P. Abrahamson J. Barlow A. Daly M. J., 1987 MAPMAKER: an interactive computer package for constructing primary genetic linkage maps of experimental and natural populations. Genomics 1: 174–181. 10.1016/0888-7543(87)90010-3 3692487

[bib41] Lee S. B. Suh M. C., 2015 Advances in the understanding of cuticular waxes in *Arabidopsis thaliana* and crop species. Plant Cell Rep. 34: 557–572. 10.1007/s00299-015-1772-2 25693495

[bib42] Leiboff S. Li X. Hu H. C. Todt N. Yang J., 2015 Genetic control of morphometric diversity in the maize shoot apical meristem. Nat. Commun. 6: 8974 10.1038/ncomms9974 26584889 PMC4673881

[bib43] Li Q. Wan J., 2005 SSRHunter: development of a local searching software for SSR sites. Hereditas 27: 808–810. 16257914

[bib44] Li Y. Fan C. Xing Y. Jiang Y. Luo L., 2011 Natural variation in *GS5* plays an important role in regulating grain size and yield in rice. Nat. Genet. 43: 1266–1269. 10.1038/ng.977 22019783

[bib45] Lichtenthaler H. Wellburn A., 1983 Determination of total carotenoids and chlorophylls a and b of leaf extract in different solvents. Biochem. Soc. Trans. 11: 591–592. 10.1042/bst0110591

[bib46] Lichtenthaler H. K., 1987 Chlorophyll and carotenoids: pigments of photosynthetic biomembranes. Methods Enzymol. 148: 350–382. 10.1016/0076-6879(87)48036-1

[bib47] Lieber M. R., 2010 The mechanism of double-strand DNA break repair by the nonhomologous DNA end-joining pathway. Annu. Rev. Biochem. 79: 181–211. 10.1146/annurev.biochem.052308.093131 20192759 PMC3079308

[bib48] Liu L. Du Y. Shen X. Li M. Sun W., 2015 *KRN4* controls quantitative variation in maize kernel row number. PLoS Genet. 11: e1005670 10.1371/journal.pgen.1005670 26575831 PMC4648495

[bib49] Liu Y. Qin L. Han L. Xiang Y. Zhao D., 2015 Overexpression of maize *SDD1* (*ZmSDD1*) improves drought resistance in *Zea mays* L. by reducing stomatal density. Plant Cell Tissue Organ Cult. 122: 147–159. 10.1007/s11240-015-0757-8

[bib50] Livak K. J. Schmittgen T. D., 2001 Analysis of relative gene expression data using real-time quantitative PCR and the 2(-Delta C(T)). Method. Methods 25: 402–408. 10.1006/meth.2001.1262 11846609

[bib51] Lolle S. J. Berlyn G. P. Engstrom E. M. Krolikowski K. A. Reiter W. D., 1997 Developmental regulation of cell interactions in the *Arabidopsis fiddlehead-1* mutant: a role for the epidermal cell wall and cuticle. Dev. Biol. 189: 311–321. 10.1006/dbio.1997.8671 9299123

[bib52] López M. Gomez E. Faye C. Gerentes D. Paul W., 2017 *zmsbt1* and *zmsbt2*, two new subtilisin-like serine proteases genes expressed in early maize kernel development. Planta 245: 409–424. 10.1007/s00425-016-2615-2 27830397

[bib53] Lu F. Romay M. C. Glaubitz J. C. Bradbury P. J. Elshire R. J., 2015 High-resolution genetic mapping of maize pan-genome sequence anchors. Nat. Commun. 6: 6914 10.1038/ncomms7914 25881062 PMC4411285

[bib54] McClintock B., 1944 The relation of homozygous deficiencies to mutations and allelic series in maize. Genetics 29: 478–502. 17247134 10.1093/genetics/29.5.478 PMC1209260

[bib55] Miura K. Ikeda M. Matsubara A. Song X. J. Ito M., 2010 *OsSPL14* promotes panicle branching and higher grain productivity in rice. Nat. Genet. 42: 545–549. 10.1038/ng.592 20495564

[bib56] Morgante M. Brunner S. Pea G. Fengler K. Zuccolo A., 2005 Gene duplication and exon shuffling by helitron-like transposons generate intraspecies diversity in maize. Nat. Genet. 37: 997–1002. 10.1038/ng1615 16056225

[bib57] Moustakas, A., S. Souchelnytskyi, C. Heldin, B. K. Sun, J. T. Lee *et al.*, 2006 *Encyclopedic Reference of Genomics and Proteomics in Molecular Medicine*, edited by B. K. Sun. Springer: Heidelberg.

[bib58] Muir C. D. Pease J. B. Moyle L. C., 2014 Quantitative genetic analysis indicates natural selection on leaf phenotypes across wild tomato species (*Solanum sect. Lycopersicon*; Solanaceae). Genetics 198: 1629–1643. 10.1534/genetics.114.169276 25298519 PMC4256776

[bib59] Ottaviani D. LeCain M. Sheer D., 2014 The role of microhomology in genomic structural variation. Trends Genet. 30: 85–94. 10.1016/j.tig.2014.01.001 24503142

[bib60] Parks M. M. Lawrence C. E. Raphael B. J., 2015 Detecting non-allelic homologous recombination from high-throughput sequencing data. Genome Biol. 16: 72 10.1186/s13059-015-0633-1 25886137 PMC4425883

[bib61] Sachs R. K. Hlatky L. R. Trask B. J., 2000 Radiation-produced chromosome aberrations: colourful clues. Trends Genet. 16: 143–146. 10.1016/S0168-9525(99)01960-5 10729825

[bib62] Saghai-Maroof M. A. Soliman K. M. Jorgensen R. A. Allard R. W., 1984 Ribosomal DNA spacer-length polymorphisms in barley: Mendelian inheritance, chromosomal location, and population dynamics. Proc. Natl. Acad. Sci. USA 81: 8014–8018. 10.1073/pnas.81.24.8014 6096873 PMC392284

[bib63] Sagi M. Fluhr R. Lips S. H., 1999 Correction: aldehyde oxidase and xanthine dehydrogenase in a flacca tomato mutant with deficient abscisic acid and wilty phenotype. Plant Physiol. 121: 315. 10.1104/pp.120.2.571 PMC59296 10364409

[bib64] Saxena R. K. Edwards D. Varshney R. K., 2014 Structural variations in plant genomes. Brief. Funct. Genomics 13: 296–307. 10.1093/bfgp/elu016 24907366 PMC4110416

[bib65] Sebat J., 2007 Major changes in our DNA lead to major changes in our thinking. Nat. Genet. 39: S3–S5. 10.1038/ng2095 17597778

[bib66] Shang Y. Yang F. Schulman A. H. Zhu J. Jia Y., 2017 Gene deletion in barley mediated by LTR-retrotransposon BARE. Sci. Rep. 7: 43766 10.1038/srep43766 28252053 PMC5333098

[bib67] Somssich M. Je B. I. Simon R., 2016 CLAVATA-WUSCHEL signaling in the shoot meristem. Development 143: 3238–3248. 10.1242/dev.133645 27624829

[bib68] Song X. J. Huang W. Shi M. Zhu M. Z. Lin H. X., 2007 A QTL for rice grain width and weight encodes a previously unknown RING-type E3 ubiquitin ligase. Nat. Genet. 39: 623–630. 10.1038/ng2014 17417637

[bib69] Springer N. M. Ying K. Fu Y. Ji T. Yeh C. T., 2009 Maize inbreds exhibit high levels of copy number variation (CNV) and presence/absence variation (PAV) in genome content. PLoS Genet. 5: e1000734 10.1371/journal.pgen.1000734 19956538 PMC2780416

[bib70] Sun L. Liu Y. Kong X. Zhang D. Pan J., 2012 ZmHSP16.9, a cytosolic class I small heat shock protein in maize (*Zea mays*), confers heat tolerance in transgenic tobacco. Plant Cell Rep. 31: 1473–1484. 10.1007/s00299-012-1262-8 22534681

[bib71] Sun S. Zhou Y. Chen J. Shi J. Zhao H., 2018 Extensive intraspecific gene order and gene structural variations between Mo17 and other maize genomes. Nat. Genet. 50: 1289–1295. 10.1038/s41588-018-0182-0 30061735

[bib72] Sun Y. Wang M. Li Y. Gu Z. Ling N., 2017 Wilted cucumber plants infected by Fusarium oxysporum f. sp. cucumerinum do not suffer from water shortage. Ann. Bot. 120: 427–436. 10.1093/aob/mcx065 28911018 PMC5591412

[bib73] Teng F. Zhai L. Liu R. Bai W. Wang L., 2013 *ZmGA3ox2*, a candidate gene for a major QTL, *qPH3.1*, for plant height in maize. Plant J. 73: 405–416. 10.1111/tpj.12038 23020630

[bib74] Turcich M. P. Bokhari-Riza A. Hamilton D. A. He C. Messier W., 1996 PREM-2, a copia-type retroelement in maize is expressed preferentially in early microspores. Sex. Plant Reprod. 9: 65–74. 10.1007/BF02153053

[bib75] Vielle-Calzada J. P. Martinez de la Vega O. Hernandez-Guzman G. Ibarra-Laclette E. Alvarez-Mejia C., 2009 The Palomero genome suggests metal effects on domestication. Science 326: 1078 10.1126/science.1178437 19965420

[bib76] Vitte C. Panaud O., 2003 Formation of solo-LTRs through unequal homologous recombination counterbalances amplifications of LTR retrotransposons in rice *Oryza sativa* L. Mol. Biol. Evol. 20: 528–540. 10.1093/molbev/msg055 12654934

[bib77] Vráblova M. Vrabl D. Hronkova M. Kubasek J. Santrucek J., 2017 Stomatal function, density and pattern, and CO_2_ assimilation in *Arabidopsis thaliana tmm1* and *sdd1–1* mutants. Plant Biol. 19: 689–701. 10.1111/plb.12577 28453883

[bib78] Wei F. Coe E. Nelson W. Bharti A. K. Engler F., 2007 Physical and genetic structure of the maize genome reflects its complex evolutionary history. PLoS Genet. 3: e123 10.1371/journal.pgen.0030123 17658954 PMC1934398

[bib79] Wright A. J. Gallagher K. Smith L. G., 2009 discordia1 and alternative discordia1 function redundantly at the cortical division site to promote preprophase band formation and orient division planes in maize. Plant Cell 21: 234–247. 10.1105/tpc.108.062810 19168717 PMC2648079

[bib80] Xing A. Gao Y. Ye L. Zhang W. Cai L., 2015 A rare SNP mutation in *Brachytic2* moderately reduces plant height and increases yield potential in maize. J. Exp. Bot. 66: 3791–3802. 10.1093/jxb/erv182 25922491 PMC4473982

[bib81] Xue W. Xing Y. Weng X. Zhao Y. Tang W., 2008 Natural variation in *Ghd7* is an important regulator of heading date and yield potential in rice. Nat. Genet. 40: 761–767. 10.1038/ng.143 18454147

[bib82] Yang K. Guo R. Xu D., 2016 Non-homologous end joining: advances and frontiers. Acta Biochim. Biophys. Sin. (Shanghai) 48: 632–640. 10.1093/abbs/gmw046 27217473

[bib84] Young T. E. Ling J. Geisler-Lee C. J. Tanguay R. L. Caldwell C., 2001 Developmental and thermal regulation of the maize heat shock protein, HSP101. Plant Physiol. 127: 777–791. 10.1104/pp.010160 11706162 PMC129251

[bib85] Yuan L. Dou Y. Kianian S. F. Zhang C. Holding D. R., 2014 Deletion mutagenesis identifies a haploinsufficient role for gamma-zein in *opaque2* endosperm modification. Plant Physiol. 164: 119–130. 10.1104/pp.113.230961 24214534 PMC3875793

[bib86] Zhang J. Peterson T., 1999 Genome rearrangements by nonlinear transposons in maize. Genetics 153: 1403–1410. 10545468 10.1093/genetics/153.3.1403 PMC1460832

[bib87] Zhang X. Wang J. Huang J. Lan H. Wang C., 2012 Rare allele of *OsPPKL1* associated with grain length causes extra-large grain and a significant yield increase in rice. Proc. Natl. Acad. Sci. USA 109: 21534–21539. 10.1073/pnas.1219776110 23236132 PMC3535600

[bib88] Zhang X. Chen X. Liang P. Tang H., 2018 Cataloging plant genome structural variations. Curr. Issues Mol. Biol. 27: 181–194. 10.21775/cimb.027.181 28885182

[bib89] Zuo W. Chao Q. Zhang N. Ye J. Tan G., 2015 A maize wall-associated kinase confers quantitative resistance to head smut. Nat. Genet. 47: 151–157. 10.1038/ng.3170 25531751

